# Analytical Approaches for the Determination of Buprenorphine, Methadone and Their Metabolites in Biological Matrices

**DOI:** 10.3390/molecules27165211

**Published:** 2022-08-16

**Authors:** Xiaoyue Shan, Chengjian Cao, Bingsheng Yang

**Affiliations:** Hangzhou Occupational Disease Prevention and Treatment Hospital, Hangzhou 310014, China

**Keywords:** analytical methods, methadone, buprenorphine, biological matrices

## Abstract

The abuse of buprenorphine and methadone has grown into a rising worldwide issue. After their consumption, buprenorphine, methadone and their metabolites can be found in the human organism. Due to the difficulty in the assessment of these compounds by routine drug screening, the importance of developing highly sensitive analytical approaches is undeniable. Liquid chromatography tandem mass spectrometry is the preferable technique for the determination of buprenorphine, methadone and their metabolites in biological matrices including urine, plasma, nails or oral fluids. This research aims to review a critical discussion of the latest trends for the monitoring of buprenorphine, methadone and their metabolites in various biological specimens.

## 1. Introduction

Opioid use disorder (OUD) is a serious public health issue [1,2]. The disorder is associated with high morbidity and mortality rates as well as an increased risk and cost of crime [3]. Approximately 26.8 million people were estimated to have OUD globally in 2016 and at least 100,000 overdose deaths are reported each year [4]. The US Food and Drug Administration (FDA) have approved buprenorphine (BUP) and methadone (MTD) for the treatment of OUD; these medications have great efficacy and can save lives [5]. BUP and MTD are synthetic opioids widely employed as analgesics to control and treat pain [6]. They are also effectively applied as medications in the treatment of OUD patients [7].

BUP is a semi-synthetic opioid possessing partial u-type agonist and k-type antagonist opioid activity [8]. As an analgesic, BUP is about 25–50 times more potent than other opioids, including morphine [9]. Several studies reported that the higher BUP dose (16–32 mg/day) is safer than a lower dose of BUP (less than 16 mg/day) for treatment [10]. Generally, patients have high rates of illicit opioid use with a lower BUP dose. BUP has a ceiling effect which reduces the risk of overdose. Doses above 32 mg/day may not increase its respiratory depressant effect [11]. The regular dose of BUP for pain relief is 0.2–0.4 mg sublingually up to 3–4 times per day [6]. BUP undergoes N-demethylation to its major metabolite norbuprenorphine (NBUP) via cytochrome P_450_3A4 [12]. Both the metabolite and the parent drug are then metabolized to buprenorphine-glucuronide (BUP-G) and norbuprenorphine-glucuronide (NBUP-G) through glucuronidation [13]. The majority of the dose (about 50–70%) is excreted through the feces, whereas approximately 10–30% of the dose is excreted in urine, mainly as conjugated metabolites. The mean excretion ratios of BUP-G and NBUP-G in the bile after administration of 0.6 mg/kg BUP were approximately 75% and 19%, while that of BUP and NBUP were less than 1% [14].

**Figure 1:**
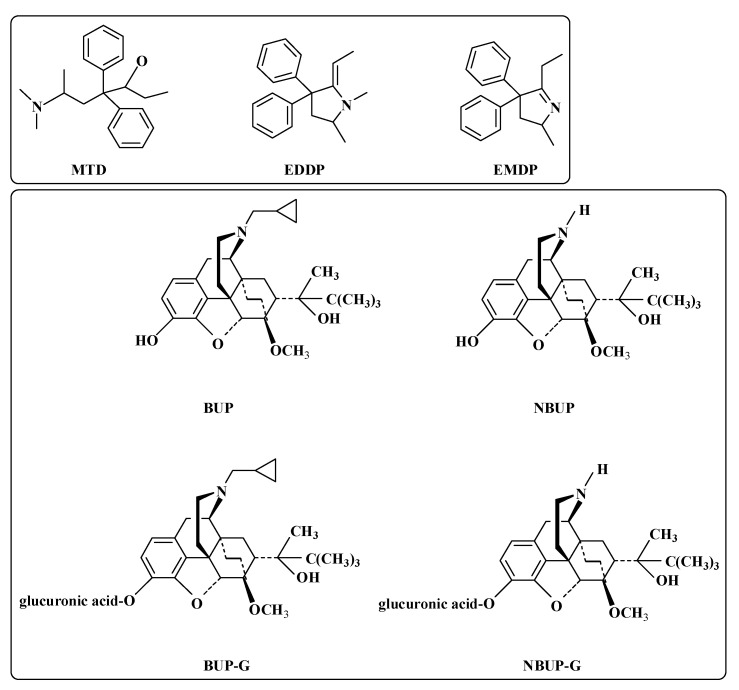
Chemical structures of BUP, MTD and their metabolites.

MTD is a synthetic opioid, which is available as a racemic mixture, with R-methadone being more potent than S-methadone [15]. The use of MTD may result in overdose because it has no ceiling effect. Doses generally started at 10–30 mg/day and gradually increased every few days, up to 80–160 mg/day [5]. MTD is regularly used in pain treatment with doses of 5–10 mg orally 3–4 times daily [6]. After oral administration, MTD is rapidly absorbed. The measurable plasma concentration is obtained after 15 to 45 min, and peak plasma concentration is achieved after 2.5 to 4.4 h [16]. MTD is metabolized to its main metabolite 2-ethylidene-1, 5-dimethyl-3,3-diphenylpyrrolidine (EDDP) by N-demethylation [17,18]. Subsequently, it is cyclized to 2-ethylidene-5-methyl-3,3-diphenylpyrrolidine (EMDP) [18]. The structures of BUP, MTD and their metabolites are shown in Figure 1.

BUP and MTD are relatively safe alternatives to opioid drugs; however, overdoses have been a common phenomenon. Excessive usage of these drugs may result in severe physical and mental injury, or even lead to death [1]. Hence, there is a high demand of developing sensitive and selective analytical methods for monitoring of BUP, MTD and their metabolites in biological matrices.

Different biological specimens including urine [7,18,19,20,21,22,23,24,25,26,27,28,29,30,31,32,33,34,35,36,37,38,39,40,41,42,43,44,45,46,47], plasma [13,20,21,35,38,40,42,43,44,45,46,47,48,49,50,51,52,53,54,55], serum [30,56,57,58,59], blood [15,18,22,49,60,61,62,63], nails [64], oral fluid [17,31,55,65,66,67,68,69], hair [47,70,71,72], tissue [73,74] and exhaled breath condensate [75,76] have been used for the measurement of BUP, MTD and their metabolites. The preferred matrix is urine, because its sampling is non-invasive, samples can be easily collected and urine samples are usually greater in quantity in comparison with other specimens [77]. The identification and quantification of BUP, MTD and their metabolites in different matrices has been challenged due to the complexity of the biological matrix and the low concentrations of these highly potent drugs. Liquid chromatography tandem mass spectrometry (LC-MS/MS) is the preferred technique due to its high selectivity and sensitivity. Furthermore, sample pretreatment is also a crucial step to remove matrix interferences and extract the target drugs.

The search was conducted on PubMed and Google Scholar databases, using the following keywords alone or in combination: “buprenorphine”, “methadone”, “metabolite”, “biological matrice” and “analytical approach”. Inclusion criteria included: (i) articles reviewing the determination of buprenorphine, methadone and their metabolites only in human biological samples; (ii) the type of article was original; (iii) articles published from 2017 to 2021; (iv) full-text articles. Exclusion criteria included: (i) articles were not peer-reviewed; (ii) non-English language; (iii) conference proceedings and editorials. This review is to offer an overview of the newest trends of analytical approaches published from January 2017 to December 2021 for the measurement of BUP, MTD and their metabolites in biological matrices. We divide the review into three sections. First, we present the commonly applied biological matrices. Second, we introduce the pretreatment procedures applied in recent studies to extract BUP, MTD and their metabolites from biological matrices. Finally, we review the analytical techniques reported for the monitoring of BUP, MTD and their metabolites in biological matrices. The review focuses on analytical methods based on the use of liquid chromatography tandem mass spectrometry (LC-MS/MS), since it is the most preferable technique for the analysis of BUP, MTD and their metabolites. Other methods including liquid chromatography (LC), gas chromatography (GC), electrochemical sensor, capillary electrophoresis (CE) and related techniques are also critically introduced, especially the main advantages and drawbacks of these methods.

## 2. Biological Matrices

Conventional biological matrices including urine, blood, plasma and serum have been used for decades for the analysis of BUP, MTD and their metabolites. Urine is the most commonly applied biological matrix likely due to the fact that the procedure is less invasive, samples can be easily collected, and sample volumes are usually larger than other matrices. However, adulteration of the urine may produce false-negative results [77]. Although the sample collection is invasive and needs medically trained staffs and requires appropriate conditions to be stable, blood (plasma, serum) is undoubtedly still widely applied in the analysis of BUP, MTD and their metabolites due to its relatively homogeneous matrix and detectable unchanged substance [78,79,80]. Nowadays, besides blood and urine, more and more unconventional biological specimens including nails, oral fluid (saliva), hair, tissue and exhaled breath condensate are applied as available alternatives to the traditional matrices.

Hair and nail sample collection are non-invasive, easy to perform and easy to store, transport and handle even under adverse conditions, decreasing the risk of sample degradation [81]. Preparation of hair and nail matrices requires several complex steps before the extraction procedure, including decontamination and homogenization [81]. The decontamination process of the hair and nail can remove the exogenous contaminants, dirt fragment and grease [82]. However, during the washing procedure, hair and nail damage may occur which can lead to the decomposition of some components. Additionally, the preparation of hair and nail matrices is laborious, time-consuming and increases the risks of errors.

Oral fluid consists of saliva and has been applied as an alternative matrix in the assessment of drugs levels. The sample collection of this matrix is quite easy, non-invasive and does not need trained professionals. Additionally, in comparison with urine or plasma, oral fluid testing is less influenced by endogenous interference. However, oral fluid analysis also has a variety of drawbacks. Generally, drug concentrations in oral fluid may be lower than concentrations in conventional biological matrices. Furthermore, drug concentrations of salivary can be highly dependent on the salivary pH and flow [17,83].

Other unconventional biological matrices like vitreous humor (VH) and skeletal tissue offer several advantages than traditional biological matrices. The sample collection of VH and skeletal tissue are easy and there are no interfering analytes that embarrass forensic toxicology testing in this matrix [84]. In addition, these matrices remain stable for a long time even after death [85]. A few studies have been reported in describing the determination of BUP, MTD and their metabolites in VH and skeletal tissue [39,74,85]. They concluded that VH and skeletal tissue are particularly important alternative matrices for post-mortem biochemical investigations.

Exhaled breath condensate has attracted substantial interest since the late 1990s. The important advantage of exhaled breath condensate is that breath collection is non-invasive, safe, non-destructive and can be collected “on demand” in a time as short as seconds [86,87]. Herein, breath analysis is excellent compared to conventional biological matrices making it a useful tool in detection.

## 3. Sample Pretreatment

A sample pretreatment before the analysis of BUP, MTD and their metabolites in biological matrices is necessary. Various sample pretreatment techniques have been reported. Simple sample pretreatment procedures like evaporation [13,72,75] and dilution [26,34,39] have been applied. Agostini et al. [26] employed a UHPLC-MS/MS technique for the analysis of BUP, NBUP, BUP-G and NBUP-G in urine samples. Urine samples were directly analyzed after dilution in water containing formic acid. This rapid and automatable method is a potential tool for routine quantification of BUP and its metabolites. Nevertheless, the most commonly employed sample pretreatment for the monitoring of BUP, MTD and their metabolites in biological matrices are liquid-liquid extraction (LLE) [18,19,20,21,22,23,26,40,47,49,50,52,53,54,56,60,61,63,65,68,69,71,74,76,77,80,88,89,90,91,92] and solid-phase extraction (SPE) [7,24,28,38,41,43,44,45,46,48,55,68,72,73,75,93,94,95]. The major benefits of LLE are its simplicity and high recovery in the monitoring of BUP, MTD and their metabolites in biological samples. Various solvents have been applied to extract BUP, MTD and their metabolites, including ethyl acetate [18,60], butyl chloride [59], hexanes or a mixture of solvents including methyl t-butyl ether/hexane (2:1, v:v) [52] or hexane/ethyl acetate (9:1) [72]. Nowadays, liquid microextraction techniques have been developed to minimize the organic solvent consumption, which decreases the risk of environmental pollution and reduces the analysis costs. Fernández et al. [67] described a UHPLC-MS/MS technique for the monitoring of 20 illegal drugs in oral fluid samples based on dispersive liquid–liquid microextraction (DLLME). The cloudy solution was formed when 200 μL of CHCl_3_ was added. Then the mixture was put in an ultrasonic bath for 5 min and centrifuged for 5 min. After that, the extraction phase was collected and dried under nitrogen flow. Finally, the sediment was redissolved in the mobile phase and injected into the GC-MS system.

Another sample preparation method for efficient preconcentration of BUP, MTD and their metabolites is SPE. SPE has received much attention due to its simplicity, short extraction time and low solvent consumption. A number of sorbents have been employed for the extraction and detection of BUP, MTD and their metabolites, including magnetic nanoparticles (NPs) [45], molecularly imprinted polymers (MIPs) [42] and metal-organic frameworks [40]. Lamei et al. [45] utilized a magnetic nanocomposite composed of Fe_3_O_4_ nanoparticles/graphene oxide/deep eutectic solvent (Fe_3_O_4_@GO-DES) to extract MTD from urine and plasma samples. The determination was carried out using both gas chromatography-mass spectrometry (GC-MS) and gas chromatography-flame ionization detector (GC-FID) to achieve the best accuracy. With this sorbent, a high preconcentration factor (PF) of 250 was obtained. Ganjavi et al. [42] synthesized a magnetic MIP for extraction of BUP in biological fluids and tablets. Owing to its high surface area and selective recognition, a high sorption capacity (76.9 mg/g) was observed. Similarly, Mohammadi et al. [40] evaluated the efficiency of metal-organic frameworks (zeolitic imidazole framework-67) for extraction and detection of BUP in biological fluids. The zeolitic imidazole framework-67 was selected due to its high extraction recovery (95–111%) for the monitoring of BUP in biological fluids.

## 4. Liquid Chromatography Hyphenated Techniques

Among chromatographic techniques, LC is considered the preferred choice for the determination of BUP, MTD and their metabolites. The benefits of HPLC for detecting BUP, MTD and their metabolites in biological samples including high selectivity, sensitivity and reproducibility. In addition, compared to GC, no complicated derivatization steps are needed. Table 1 summarizes the details based on the use of LC techniques utilized for BUP and MTD measurement. LC can be coupled with ultraviolet (UV) [40,41,42,43,54], photodiode array detector (PAD) [40], fluorescence (FL) and electrochemical (EC) [29,38] MS detection [7,13,15,18,23,24,25,26,27,28,49,50,52,56,60,61,62,64,65,68,69,70,71,72,73,88]. Reversed-phase columns (C_18_ or C_8_ columns) with spherical sorbent particles are popular for the determination of BUP, MTD and their metabolites. The limits of detections (LODs) of the methods are extremely low, which is due to the high sensitivity of detectors and high selectivity of the sorbents. In a study, Ganjavi et al. [43] synthesized MIPs for the selective extraction of BUP from plasma and urine by LC-UV. Due to its good selectivity and high adsorption capacity, the method exhibits excellent clean up properties. The advantages of the method included wide linear dynamic range (LDR), low limit of detection (3.0 μg/L), good precision and a high PF. In a similar work, Habibi et al. [94] applied magnetic MIP NPs for dispersive magnetic solid-phase extraction (DMSPE) of BUP in human urine samples, followed by LC-FL analysis. After MSPE, the LOD and recovery of the method achieved 0.21 µg/L and 97.4–100.3%, respectively. The method tended to be a potential and innovative sample treatment and detection method in routine drug analysis.

In a clinical study, LC with an EC was utilized to monitor MTD in the blood samples [59]. The low LOD of 0.5 ng/mL was comparable to the LC/MS/MS method. The method was specific enough for the monitoring of serum MTD levels in cancer patients. Gomar et al. [54] proposed a LC-UV method coupled with MSPE for the monitoring of BUP and its metabolites in human plasma samples. The MSPE technique was based on a poly para-phenylenediamine modified Fe_3_O_4_ NPs (PpPDA/Fe_3_O_4_), which greatly improved the extraction efficiency (>90%) and decreased the analysis time (<20 min).

UHPLC is usually recognized as an alternative to current LC, owing to its higher separation efficiency and shorter analysis time. Mohammadi et al. [40] proposed a UHPLC method for the monitoring of BUP in biological fluids after dispersive SPE sample pretreatment with zeolitic imidazole framework-67. The instrument was equipped with a PDA and a UV detector. This method obtained a very low LOD of 0.15 µg/L and a small consumption of organic solvent (1.9 mL). Akhgari et al. [39] demonstrated a UHPLC-PDA method for the quantification of MTD and tramadol in postmortem VH samples after DLLME sample pretreatment with chloroform and methanol. The developed method exhibited a low LOD (3 µg/L) and high levels of accuracy (99.4–100%) for MTD analysis. The method was adequate for the monitoring of MTD and tramadol in forensic toxicology analysis.

Tandem mass spectrometry (MS/MS) and high-resolution mass spectrometry (HRMS) were widely applied in the identification and quantification of analytes due to their high sensitivity, which usually may achieve ultra-trace concentration levels. Mariottini et al. [7] described an automated SPE coupled with LC-MS/MS method for the quantification of BUP, naloxone (NLX) and their metabolites in urine samples. The LODs of these compounds were ranged from 0.3 to 1.0 µg/L. Chan et al. [90] reported the simultaneous monitoring of BUP and NBUP in whole blood samples by LC-MS/MS method. The LOQs of BUP and NBUP were 4.4 and 3.4 µg/L, respectively. The blood samples were extracted with a mixture of ethyl acetate and cyclohexane, then centrifuged at 6000 rpm for 10 min, injected into a Kinetex 5u C_18_ 100A column and eluted with a mixture of acidic acetonitrile, methanol and acidic water mobile phase. The fast UHPLC separation can be utilized to monitor the multi-analyte mixtures in biological samples. Application of UHPLC-HRMS technique for the measurement of 16 opioids and derivatives, including BUP, NBUP, MTD and EDDP after LLE sample preparation with zinc sulfate, methanol and acetonitrile was developed by Feliu et al. [61]. The method required only 100 µL of the blood sample. Fast analysis and acquisition time (5.10 min) was achieved using a UHPLC Waters Acquity HSS T3 column (50 mm × 2.10 mm, 1.8 µm) at 50 °C with a gradient composed of water (containing 0.1% formic acid) and acetonitrile (containing 0.1% formic acid). The retention times of BUP, NBUP, MTD and EDDP were 3.48, 3.3, 3.59 and 3.53, respectively. The LODs obtained ranged from 0.1 to 0.5 µg/L. In another study, a LC-MS method was utilized for the measurement of MTD, COC and methamphetamine (MTA) in oral fluid after the microextraction technique with a nylon 6 modified wooden toothpick (N6-WT) [65]. The LOD was 0.5 µg/L for MTD. These low LODs were obtained owing to the microextraction techniques applied in the sample pretreatment and the high-sensitivity detector. However, Agostini et al. [26] proposed the use of the automatable UHPLC-MS/MS method for the measurement of BUP and its metabolite in urine samples without any sample pretreatment. The diluted urine samples were directly analyzed by UHPLC-MS/MS. The obtained LODs of BUP and its metabolite were as low as 0.5–1.5 µg/L. The retention times of BUP and NBUP were 4.44 and 3.69, respectively. In addition, the method was applied to 30 real urine samples. The metabolic ratio calculated as NBUP/BUP gave an idea of the intake time. For example, NBUPtot/BUPtot ratio equal to 1 indicated 7–10 h after BUP intake. The combination of UHPLC and MS/MS greatly improves the specificity and separation speed. However, it also has drawbacks such as high cost and large size of the instruments. Other applications of LC-MS/MS or LC-HRMS technique for the monitoring of BUP, MTD and their metabolites in biological samples have also been listed in Table 1.

## 5. Gas Chromatography Hyphenated Techniques

GC methods applied for the measurement of MTD, BUP and their metabolites are summarized in Table 2. In most of the studies, GC is not as widely utilized as LC for the quantitation of BUP, MTD and their metabolites in biological matrices. As GC can only work with volatile and semi-volatile constituents, a complex derivatization step prior to GC analysis is required, resulting in an increase of the overall time required to analyze the targets. To reduce this time, Lin et al. [91] established a GC-MS method coupled with a UADLLME pretreatment procedure for the monitoring of seven recreational drugs (including MTD) in human blood samples, without derivatization. The UA-DLLME procedure was conducted by using methanol as the dispersing solvent and dichloromethane as the extraction solvent. The main advantages of this UADLLME method were the low consumption of dichloromethane (200 µL) and blood volume (200 µL), making it available for forensic cases. Lamei et al. [45] reported a pretreatment method based on MSPE for monitoring of MTD in urine and plasma samples. A new adsorbent was prepared by coating a new deep eutectic solvent onto the magnetic GO surface, which was donated as Fe_3_O_4_@GO-DES. The synthesized Fe_3_O_4_@GO-DES was used for efficient extraction of MTD. Finally, both GC-FID and GC-MS were applied to detect the MTD. The obtained LODs of the method were 0.8 µg/L for GC-FID and 0.03 µg/L for GC-MS. In addition, the high PF was 250.

Besides urine, blood and plasma samples, GC-MS analysis of MTD, BUP and their metabolites in oral fluid has also been reported. Oral fluid testing is non-invasive and less influenced by endogenous interference. Thus, it is more desirable for detecting MTD and BUP in patients. For example, Shekari et al. [89] used an ultrasound assisted DLLME (UADLLME) as a sample pretreatment method for MTD measurement in saliva. The saliva samples were extracted by sodium hydroxide and chloroform and held at ultrasonic bath before introduced into GC-MS analysis. The researchers also investigated the DLLME/GC-MS method for the detection of MTD in saliva, which required more extraction solvent and provided lower recovery in comparison with the UADLLME/GC-MS method. Ribeiro et al. [17] described the utilization of GC-MS/MS for the monitoring of MTD and EDDP in oral fluid samples. The dried saliva spots (DSS) were applied for the collection and preservation of oral fluid samples. The extraction procedure was performed using isopropanol as organic solvent, subsequently analyzed by GC-MS/MS system. With the utilization of DSS and GC-MS/MS, the method was successfully applied in the quantification of MTD and EDDP in oral fluid samples from patients undergoing MMT. In another work, Ezoddin et al. [55] proposed an ultrasonic-assisted supramolecular model based on solidification of floating organic drop microextraction (UA-SM-SFO-ME) for the preconcentration of MTD in plasma and saliva samples before GC-MS analysis. A mixture of 1-Dodecanol and THF were selected as supramolecular solvents for the microextraction of MTD, which may be an appropriate alternative to organic solvents owing to its low toxicity. Moreover, the major advantage of this method was the application of ultrasonication, which provided fast extraction, high extraction recovery and low detection limit.

## 6. Other Techniques

Besides the analytical approaches based on LC and GC, other analytical techniques developed for the determination of MTD, BUP and their metabolites have also been reported, including capillary electrophoresis (CE) [46,76], electrochemical sensor [29,30,31,32,33,34,35,36,37,38,53,57,58,63], enzyme immunoassay [22], as shown in Table 3. Among these analytical approaches, electrochemical method is the most popular one due to its high sensitivity, low cost and fast response time. The citrate stabilized magnetic nanocrystals (CS-MNCs) were coated onto the surface of the carbon paste electrode (CPE) by the Farmany’ group [95]. The synthesized CS-MNCs/CPE was applied for the quantification of BUP in human plasma and urine samples. The sensor obtained a LOD of 4.3 nM. The advantages of the method were high sensitivity, simplicity, speed and no sample pretreatment or separation procedure was required. Similarly, Alizadeh et al. [34] described a MIP and multiwalled carbon nanotubes (MWCNT) modified CPE for monitoring of BUP in human urine samples without the need for pretreatment. The combination of MWCNT and MIP significantly increased the sensitivity and selectivity of the CPE. Under optimum conditions, the sensor achieved a LOD of 0.6 nM. In addition, the sensor has the potential for determination of other semi structure drugs.

CE and related techniques have also been effective approaches for the measurement of MTD, BUP and their metabolites due to their cost-effectiveness, automation, simplicity and less sample consumption. For example, Naghdi et al. [46] reported the use of a maltodextrin modified CE for the chiral analysis of MTD and tramadol (TRA) in tablet, urine and plasma samples. Under optimal extraction conditions, the method achieved LODs of 2 µg/mL for TRA and 1.5 µg/L for MTD. Later, Cui et al. [96] employed the CE method in combination with PAD detection for determining 46 drugs of abuse, including MTD and BUP in whole blood samples. The obtained LODs were both 30 µg/L for MTD and BUP. Finally, the method was utilized to detect real blood samples in forensic investigation. These data demonstrated that the lack of sensitivity of CE-based techniques is a main contributing factor to its limited application.

An interesting identification method was developed by Farquharson et al. [96], who described a rapid quantitation method based on surface-enhanced Raman spectroscopy (SERS) for BUP and opioids detection in saliva. A simple liquid extraction was carried out to extract the BUP from saliva. The analysis was in good agreement with the urinalysis result and most importantly, the analysis time was only 25 min. We also notice that there is a method based on enzyme immunoassay by Forouzesh’s group [22]. This is the study performed to compare two methods, ELISA and GC-MS, for measuring MTD levels. Both methods were acceptable. Nevertheless, in GC-MS the analysis range was from 30 ng–10 µg, while in ELISA the linearity was much lower, from 1.2 ng–100 ng. In general, GC-MS is preferable to ELISA due to its high sensitivity; however, ELISA can detect a large number of samples in a short time with rapid speed.

## 7. Conclusions

The monitoring of BUP, MTD and their metabolites in various biological specimens has been a challenging task in clinical and forensic toxicology. Unconventional biological specimens are emerging as available alternatives because they are non-invasive, easy to perform and non-destructive, while ensuring accuracy and sensitivity at the same time. Owing to the complexity of matrices, analytical procedures usually consist of sample pretreatment steps and highly sensitive instrumentation analysis. Simple LLE or SPE, especially the microextraction techniques, are commonly applied to minimize matrix interferences and maximize the target concentrations. Chromatographic techniques including LC, GC or the combination of HRMS or MS with LC or GC are applied to obtain ultra-trace concentration levels. The approaches based on LC-MS/MS are the preferable techniques for the quantitation of BUP, MTD and their metabolites in biological samples. Moreover, other methodologies are also reported by researchers, including electrochemical methods and CE-based techniques. Taking into account the serious abuse of BUP and MTD, the measurement of these compounds and their metabolites in biological matrices are worth further exploration.

## Figures and Tables

**Table 1:** LC techniques applied for the analysis of BUP, MTD and their metabolites in biological samples.

Target Analytes	Matrices	Techniques	Extraction	Mobile Phase	LOD (LOQ or LLOQ)	Ref.
BUP, NAL, their metabolites	Urine	LC-MS/MS (Gemini-NX C18, 100 mm × 2.1 mm, 4 μm; C18 guard column, 4 mm × 2 mm)	SPE	Methanol and ammonium acetate buffer (both containing 0.1% formic acid)	BUP: 0.3 µg/L, NAL: 0.5 µg/L, NAL-G: 1 µg/L, NBUP: 1 µg/L, BUP-G: 0.3 µg/L, NBUP-G: 1 µg/L	[7]
BUP, NAL, their metabolites	Plasma	LC-MS/MS (Thermo HILIC, 100 mm × 2.1 mm, 3.5 μm)	LLE	60% MeCN and 40% aqueous 25m M ammonium formate (pH 3.5)	/	[13]
MTD, EDDP	Post-mortem samples	LC-MS/MS SFC-MS/MS (AGP, 100 mm × 2.1 mm, 5 μm; 10 × 2.0 mm; 5 μm)	SPE	10 mM Ammonium acetate(pH 5.8) and isopropanol	MTD: 2.5 µg/L in LC, 0.5 µg/L in SFC	[15]
MTD, EDDP, EMDP	Postmortem Matrices	LC-MS/MS (Kinetex XB-C18, 150 mm × 2.1 mm, 2.6 μm)	LLE	/	LLOQ: MTD: 0.5 µg/L, EDDP: 0.5 µg/L, EMDP: 0.5 µg/L	[18]
BUP, NBUP, NAL	Urine	LC-MS/MS (CORTECS Phenyl, 50 mm × 2.1 mm, 1.6 μm)	SPE	0.05% Formic acid in water and 0.1% formic acid in acetonitrile	LLOQ: NBUP: 5 µg/L	[23]
MTD, BUP and other durgs	Urine	UHPLC-MS/MS (HSS T3, 100 mm × 2.1 mm, 1.8 μm)	LLE	Methanol and 5 mM ammonium acetate containing 0.025% formic acid in water	BUP: 2 µg/L, NBUP: 2 µg/L, MTD: 1 µg/L, EDDP: 0.5 µg/L	[25]
BUP, NBUP and their metabolites	Urine	UHPLC-MS/MS (PFP, 50 mm × 2.1 mm, 1.9 μm)	/	95% ACN with 0.1% formic acid and 5% formic acid	BUP: 0.5 µg/L, NBUP: 1.5 µg/L, NBUP-G: 0.5 µg/L, BUP-G: 1.0 µg/L	[26]
16 Drugs	Urine	LC-MS (XDB C18, 150 mm × 2.1 mm, 5 μm)	SPE	Ultra-pure water/0.1% HCOOH and MeOH/0.1% HCOOH	MTD: 5 µg/L, EDDP: 20 µg/L	[27]
BUP, NBUP	Urine	LC-MS/MS	/	/	BUP: 0.5µg/L, NBUP: 0.5µg/L	[28]
MTD, tramadol	Vitreous Humor	UPLC-PDA (C18, 150 mm × 3 mm)	DLLME	Phosphate buffer (pH = 2.32) and acetonitrile	MTD: 3 µg/L	[39]
BUP	Serum	UPLC-PDA/UV (C18, 250 mm × 4.6 mm, 5 μm)	SPE	95% Methanol and 5% deionized water containing 4 mM 1-octane sulfonic acid	0.15 µg/L	[40]
BUP	Plasma, urine, tablets	LC-UV (ODS-H C18, 150 mm × 4.6 mm, 5 μm)	MSPE	0.01 M Phosphate buffer (pH 3.1) and acetonitrile	0.6 µg/L	[42]
BUP	Plasma, urine	LC-UV (ODS-H C18, 150 mm × 4.6 mm, 5 μm)	SPE	Acetonitrile and 0.01 M phosphate buffer with pH 3.1	3 µg/L	[43]
MTD, EDDP	Dried blood spots	LC-MS/MS (Chiral-AGP, 150 mm × 4.6 mm, 5 μm)	LLE	Acetonitrile (gradientfrom10 to34%) in 0.1% formic acid (pH 6.5)	/	[49]
BUP	Plasma	LC-MS/MS (XB-C18, 50 mm × 2.1 mm, 2.6 μm)	LLE	0.1% Formic acid and methanol	0.25 µg/L	[50]
BUP, NBUP	Plasma	LC-UV (Nova-pak C18, 250 mm × 4.6 mm, 5 μm)	MSPE	Phosphate buffer (pH 3.4) and acetonitrile	BUP: 0.8 µg/L, NBUP: 0.3 µg/L	[54]
BUP, gabapentin	Serum	LC-MS/MS (Biphenyl 100Å, 50 mm × 2.1 mm, 5 μm)	LLE	10 mM Ammonium formate and methanol containing 0.1% formic acid	BUP: 1 µg/L	[56]
MTD	Serum	LC-ECD (RP18, 50 mm × 4.6 mm, 5 μm)	LLE	10 mM Na2HPO4, CH3CN and CH3OH	0.5 µg/L	[59]
MTD, BUP and their metabolites	Blood	UHPLC-MS-MS (BEH C18, 150 mm × 2.1 mm, 1.7 μm)	LLE	0.1% Formic acid in water and 0.1% formic acid in methanol	MTD: 0.41 µg/L, EDDP: 1.41 µg/L, BUP: 0.59 µg/L, NBUP: 0.66 µg/L,	[61]
MTD, BUP, EDDP and other opioids	Whole blood	UPLC-HRMS (HSS T3, 50 mm × 2.1 mm, 1.8 μm)	LLE	0.1% Formic acid in water and 0.1% formic acid in acetonitrile	BUP: 0.15 µg/L, NBUP: 0.1 µg/L, MTD: 0.5 µg/L, EDDP: 0.5 µg/L	[62]
MTD, EDDP	Dried blood spots and plasma	LC-MS/MS (Eclipse XDB, 12.5 mm × 4.6 mm, 5 μm)	/	0.1% Formic acid in water and methanol	LLOQ: MTD: 0.1 µg/L, EDDP: 0.1 µg/L, EMDP: 0.1 µg/L	[63]
MTD, COC, methamphetamine	Oral fluid	LC-MS/MS	SPME	0.1% Ammonium formate aqueous solution	MTD: 1.5 µg/L	[66]
MTD, EDDP and other 15 drugs	Oral fluid	LC-MS/MS (Hypersil PFP, 50 mm × 2.1 mm, 1.9 μm)	/	0.1% Formic acid in water and 0.1% formic acid in methanol/AcN	/	[67]
20 Drugs	Oral fluid	UPLC-MS/MS (RP 18, 100 mm × 2.1 mm, 1.7 μm)	UADLLME	0.1% Formic acid in water and 0.1% formic acid in acetonitrile	BUP: 1 µg/L, MTD: 0.1 µg/L, EDDP: 0.5 µg/L	[68]
Novel synthetic opioids, morphine and BUP	Oral fluid	LC-MS/MS (EC-C18, 100 mm × 3.0 mm, 2.7 μm; 2.1 mm× 5.0 mm, 2.7 μm)	SPE	0.05% Formic acid, 5mM ammonium formate in water and 0.1% formic acid in acetonitrile	BUP: 5 µg/L	[69]
21 Drugs	Oral fluid	UHPLC-MS/MS (RP 18, 100 mm × 2.1 mm, 1.7 μm)	MEPS	0.1% Formic acid in water and 0.1% formic acid in acetonitrile	LOQs: 0.5–1 µg/L	[70]
60 Drugs	Hair	UHPLC-HRMS/MS (PFP, 100 mm × 2.1 mm, 2.6 μm)	DLLME	0.1% Formic acid in water and 0.1% formic acid in ace tonitrile/methanol	BUP: 2 pg/mg, NBUP: 2 pg/mg, MTD: 0.2 µg/L, EDDP: 0.5 pg/mg	[71]
MTD, EDDP, EMDP	Skeletal tissue	LC-MS/MS (BEH C18, 50 mm × 2.1 mm, 1.7 μm)	LLE	An aqueous buffer (pH 4) and acetonitrile	MTD: 0.1 ng/g, EDDP: 0.17 ng/g, EMDP: 0.11 ng/g	[74]
BUP, MTD, oxycodone, fentanyl, tramadol	Postmortem Matrices	UPLC-MS/MS	LLE	/	LLOQ: MTD: 0.011µg/mL, BUP: 0.94 µg/L	[75]
28 Drugs	Exhaled breath	LC-MS/MS (BEH phenyl, 100 mm × 2.1 mm, 1.7 μm)	LLE	5% Methanol in water with 4 mM ammonium formate and 5% methanol in water with 0.1% ammonia	MTD: 1.2 pg/filter, EDDP: 0.5 pg/filter, BUP: 4 pg/filter, NBUP: 10 pg/filter	[76]
40 Drugs	Breast milk	LC-MS/MS (RP18, 125 mm × 2.0 mm, 5 μm)	LLE	Acetonitrile and water containing 20 mM formic acid/ammonium formate buffer (pH 3.8)	MTD: 0.5 µg/L, EDDP: 0.2 µg/L	[88]
BUP, NBUP	Whole blood	LC-MS/MS	LLE	0.1% Formic acid in acetonitrile, methanol and 0.1% formic acid in water	BUP: 4.4 µg/L, NBUP: 3.4 µg/L	[96]

**Table 2:** GC techniques applied for the analysis of BUP, MTD and their metabolites in biological samples.

Target Analytes	Matrices	Techniques	Extraction	LOD (LOQ or LLOQ)	Ref.
MTD, EDDP	Oral fluid	GC-MS/MS (30 m × 0.25 mm, 0.25 μm)	LLE	MTD: 5 µg/L EDDP: 5 µg/L	[17]
MTD, TRM	Urine	GC-FID (HP-5, 30 m × 0.25 mm, 0.25 μm)	LLME	MTD: 2.4 µg/L	[19]
MTD	Urine, plasma, saliva	GC-FID/MS (DB 5-ms, 30 m × 0.25 mm, 0.25 μm)	DLLME	GC-FID: Urine: 2.7 µg/L Plasma, saliva: 9.5 µg/L GC-MS: Urine: 0.06 µg/L Plasma, saliva: 0.2 µg/L	[20]
MTD	Plasma, urine, saliva	GC-FID (HP-5, 30 m × 0.32 mm, 0.25 μm)	/	Urine: 0.5 µg/L, Plasma: 0.7 µg/L Saliva: 1.5 µg/L	[21]
MTD, TRM	Urine, plasma, saliva	GC-MS (HP-5, 30 m × 0.25 mm, 0.25 μm)	SPE	Urine MTD: 0.45 µg/L MTD: 2.5 µg/L MTD: 0.8 µg/L	[44]
MTD	Urine, plasma	GC-FID/MS (HP-5, 30 m × 0.25 mm, 0.25 μm)	MSPE	GC-FID: MTD: 0.8 µg/L GC-MS: MTD: 0.03 µg/L	[45]
MTD, COD	Plasma	GC-FID (BP-5, 30 m × 0.25 mm, 0.25 μm)	LLE	MTD: 15 µg/L	[48]
MTD	Plasma and saliva	GC-MS (HP-5, 30 m × 0.32 mm, 0.25 μm)	UA-SM-SFO-ME	Plasma: 1.2 µg/L Saliva: 0.7 µg/L	[55]
MTD	Nail	GC-MS (VF-5ms, 30 m × 0.32 mm, 0.25 μm)	LLE and SPE	MTD: 3.3 ng/mg EDDP: 6.0 ng/mg EMDP: 6.0 ng/mg	[64]
MTD, EDDP	Hair	GC-MS/MS (Capillary Column, 30 m × 0.25 mm, 0.25 μm)	MEPS	LLOQ: MTD: 0.01 ng/mg EDDP: 0.01 ng/mg	[71]
MTD, EDDP, 8 new psychoactive substances (NPS)	Hair	GC-MS (DB-5, 30 m × 0.25 mm, 0.25 μm)	LLE	MTD: 0.2 ng/mg EDDP: 0.05 ng/mg	[72]
MTD	Urine	GC-MS (HP-5MS, 30 m × 0.25 mm, 0.25 μm)	UALLE	2.1 µg/L	[88]
MTD	Saliva	GC-MS (HP-5MS, 30 m × 0.25 mm, 0.25 μm)	UADLLME	50 µg/L	[89]
7 recreational drugs	Whole blood	GC-MS (HP-5MS, 30 m × 0.25 mm, 0.25 μm)	UADLLME	MTD: 10 µg/L	[91]

**Table 3:** Other techniques applied for the analysis of BUP, MTD and their metabolites in biological samples.

Analytical Techniques	Target Analytes	Matrices	Sample Pretreatment	LOD (LOQ or LLOQ)	Ref.
Enzyme immunoassay (ELISA)	MTD	Serum	Alkaline extraction with ethyl acetate	0.18 µg/L	[21]
Electrochemical sensor	BUP	Urine	Dilution with PBS	28 nM	[29]
Electrochemical sensor	MTD	Blood serum, urine	Deproteinization with methanol	14 nM	[30]
Electrochemical sensor	MTD, morphine	Blood, urine, saliva	Dilution with PBS	MTD: 5.6 nM	[31]
Electrochemical sensor	MTD, morphine	Urine	Dilution with buffer	MTD: 3 nM	[33]
Electrochemical sensor	BUP	Urine	Dilution with britton buffer	0.6 nM	[34]
Electrochemical sensor	MTD	Serum, urine	Deproteinization with methanol	14 nM	[35]
Electrochemical sensor	MTD	Urine	Direct immersion-solid phase microextraction	0.2 µg/L	[37]
Electrochemical sensor	MTD	Blood serum, urine	Deproteinization with trichloroacetic acid	0.03 µM	[38]
Capillary electrophoresis	MTD, TRM	Urine, plasma	Dilution with water	MTD: 1.5 µg/L	[46]
Capillary electrophoresis	MTD, EDDP	Plasma	LLE with dichloromethane	LOQ: MTD: 25 µg/L EDDP: 2.5 µg/L	[53]
Electrochemical sensor	MTD	Blood	Deproteinization with 0.5 M sulfuric acid	0.12 µM	[57]
Surface-enhanced Raman spectroscopy (SERS)	BUP	Saliva	Liquid extraction with dichloromethane	/	[92]
Capillary electrophoresis	MTD	Exhaled breath Condensate, serum and urine	LLE with acetonitrile,	LLOQ: 0.15 µg/mL	[76]
Electrochemical sensor	BUP	Serum, urine	Deproteinization with methanol	4.3 nM	[95]
Capillary electrophoresis	46 drugs	Whole blood	SPE with an Oasis HLB column	MTD: 30 µg/L BUP: 30 µg/L	[96]

## Data Availability

Data sharing is not applicable to the paper; all supporting data are included within the main article.

## Abbreviations

OUD	Opioid use disorder
FDA	Food and Drug Administration
BUP	Buprenorphine
MTD	Methadone
NBUP	Norbuprenorphine
BUP-G	Buprenorphine-glucuronide
NBUP-G	Norbuprenorphine-glucuronide
EDDP	2-Ethylidene-1, 5-dimethyl-3,3-diphenylpyrrolidine
EMDP	2-Ethylidene-5-methyl-3,3-diphenylpyrrolidine
LC-MS/MS	Liquid chromatography tandem mass spectrometry
LC	Liquid chromatography
GC	Gas chromatography
CE	Capillary electrophoresis
VH	Vitreous humor
UHPLC-MS/MS	Ultra-high-performance liquid chromatography-tandem mass spectrometry
LLE	Liquid-liquid extraction
SPE	Solid-phase extraction
DLLME	Dispersive liquid-liquid microextraction
GC-MS	Gas chromatographymass spectrometry
NPs	Nanoparticles
MIPs	Molecularly imprinted polymers
Fe_3_O_4_@GO-DES	Fe_3_O_4_ nanoparticles/graphene oxide/deep eutectic solvent
GC-FID	Gas chromatography-flame ionization detector
PF	Preconcentration factor
UV	Ultraviolet
PAD	Photodiode array detector
FL	Fluorescence
EC	Electrochemical
LDR	Linear dynamic range
LOD	Limit of detection
DMSPE	Dispersive magnetic solid-phase extraction
LOQ	Limit of quantification
LLOQ	Lower limit of quantification
COC	Cocaine
MMT	Methadone maintenance treatment
PpPDA/Fe_3_O_4_	Poly para-phenylenediamine modified Fe_3_O_4_ NPs
MS/MS	Tandem mass spectrometry
HRMS	High-resolution mass spectrometry
NLX	Naloxone; MTA: Methamphetamine
N6-WT	Nylon 6 modified wooden toothpick
UADLLME	Ultrasound-assisted DLLME
DSS	Dried saliva spots
SHS-HLLME	Switchable hydrophilicity solvent-based homogenous liquid-liquid microextraction
Fe@GO-DES	Magnetic graphene nanoparticles coated with a new deep eutectic solvent
CSDF-ME	Continuous sample drop flow microextraction
UA-SM-SFO-ME	Ultrasonic-assisted supramolecular based on solidification of floating organic drop microextraction
UA-SM-SFO-ME	Ultrasonic-assisted supramolecular based on solidification of floating organic drop microextraction
CS-MNCs	Citrate stabilized magnetic nanocrystals
CPE	Carbonpaste electrode
MWCNT	Multiwalled carbon nanotubes
ELISA	Enzymeimmunoassay
SERS	Surface-enhanced Raman spectroscopy
TRA	Tramadol
